# Broadband optical ultrafast reflectivity of Si, Ge and GaAs 

**DOI:** 10.1038/s41598-020-74068-y

**Published:** 2020-10-15

**Authors:** A. Di Cicco, G. Polzoni, R. Gunnella, A. Trapananti, M. Minicucci, S. J. Rezvani, D. Catone, L. Di Mario, J. S. Pelli Cresi, S. Turchini, F. Martelli

**Affiliations:** 1 Physics Division, School of Science and Technology, University of Camerino, 62032 Camerino, Italy; 2 INFN, Sezione di Perugia, 06123 Perugia, Italy; 3 Istituto di Struttura della Materia (ISM), CNR, 00133 Rome, Italy; 4 Istituto per la Microelettronica e i Microsistemi (IMM), CNR, 00133 Rome, Italy

**Keywords:** Supercontinuum generation, Ultrafast photonics, Condensed-matter physics, Optics and photonics, Physics

## Abstract

Ultrafast optical reflectivity measurements of silicon, germanium, and gallium arsenide have been carried out using an advanced set-up providing intense subpicosecond pulses (35 fs FWHM, $\lambda$ = 400 nm) as a pump and broadband 340–780 nm ultrafast pulses as a white supercontinuum probe. Measurements have been performed for selected pump fluence conditions below the damage thresholds, that were carefully characterized. The obtained fluence damage thresholds are 30, 20.8, 9.6 mJ/$\hbox {cm}^2$ for Si, Ge and GaAs respectively. Ultrafast reflectivity patterns show clear differences in the Si, Ge, and GaAs trends both for the wavelength and time dependences. Important changes were observed near the wavelength regions corresponding to the $E_1$, $E_1+\Delta$ singularities in the joint density of states, so related to the peculiar band structure of the three systems. For Ge, ultrafast reflectivity spectra were also collected at low temperature (down to 80 K) showing a shift of the characteristic doublet peak around 2.23 eV and a reduction of the recovery times.

## Introduction

Semiconductor devices form the basis of nearly all applications in electronics and optoelectronics and for this reason understanding ultrafast properties of those basic materials used for advanced applications is extremely important. In particular, ultrafast optical measurements of the carrier dynamics can lead to deeper understanding of the fundamental mechanisms of the excitation and relaxation phenomena at typical $10^{-14}$ to $10^{-9}$ seconds time scales. Many efforts have been devoted to the realization of experiments on different semiconducting materials in the last decades, and important results have been obtained using ultrafast laser sources and developing suitable interpretation schemes (see for example^1–4^ and refs. therein). Studies of the ultrafast carrier dynamics have been carried out for simple semiconductors^1,4–7^ and nano-sized systems relevant for devices and applications (see for example^8–11^ and refs. therein). Recently, there is also a growing interest in pump–probe ultrafast experiments carried out at free-electron-laser facilities which have extended the available photon energy range for experiments aimed at studying the optical response of specimens using high-energy density pump pulses (see for example Refs.^12,13^ and refs. therein).

In the last decades, setups for ultrafast optical measurements (see for example Refs.^8,14,15^) have been becoming increasingly available in many laboratories worldwide. Nonetheless, systematic measurements under similar pump–probe conditions have been rarely reported, even for simple materials such as silicon, germanium or gallium arsenide, subjects of the present study. In this work, we present a set of ultrafast pump–probe reflectivity experiments on Si, Ge and GaAs bulk single-crystals at selected pump fluences (pump wavelength 400 nm) coupled with a broadband optical probe, for typical 0–300 ps delay times between pump and probe pulses. Silicon is the most well-studied example, and several publications provide a quite complete picture of the phenomenology related to its ultrafast reflectivity response. In particular, diffusion of electrons and holes as a function of carrier density for crystalline silicon have been evaluated looking at the optical transient reflectivity^6,16^, for which different contributions (free carrier, state filling and lattice heating) were identified and studied^7^. More recently, data and interpretation were extended for silicon to a wider optical range^4^. Several transient reflectivity works^5,15,17,18^ regarded also GaAs and specific models taking into account the filling of valence band, band modification and the free carrier absorption were developed^19,20^. To the best of our knowledge, there are no previous pump–probe reflectivity works on germanium, but time-resolved dielectric function data in the visible range^21^ and models for the interpretation have been proposed^22,23^. All those previous studies on Si, Ge, GaAs have been performed generally using different pump and probe wavelengths, pulsewidths, fluences, so a general picture of their ultrafast properties under conditions similar to the present work is still lacking.

The experiments presented in this work were performed with the aim to compare the ultrafast response of Si, Ge and GaAs under similar experimental pump–probe conditions. The three materials are known to present distinct band-energy features that are expected to influence the recovery dynamics as a function of the probe wavelengths. Ultrafast reflectivity spectra were thus obtained for Si, Ge and GaAs at room temperature. Measurements were extended at low temperatures for Ge (80, 150 K) where we expect distinct variations of the reflectivity features.

The static optical properties of the three materials^24^ are rather different in the investigated range. Direct consequences are thus expected both for the absorption of the pump pulses (400 nm) and for the subsequent broadband transient optical reflectivity. The three semiconductors present distinct features, such as peaks or doublets in the optical reflectivity corresponding to the $E_1$, $E_1+\Delta$ singularities in the joint density of states (following the notations of Cardona et al.^25,26^). Those features appear at different photon wavelengths 365 nm (3.4 eV, Si), 530–585 nm (2.34–2.12 eV, Ge), 400–428 nm (3.1–2.9 eV, GaAs) and correspond to interband transitions among nearly-parallel conduction and valence bands^27,28^. Moreover, the larger absorption coefficient of Ge and GaAs (represented by a non-negligible imaginary part of the refractive index *k*) at optical wavelengths (see for example Ref.^24^) is related to a reduced interaction volume (and higher energy density) of pump pulses with the samples. Therefore, a first stage of the investigation done in this work has been the determination of the ablation or damage thresholds (fluence corresponding to permanent damage) of the samples for ultrafast 400 nm pulses. The damage threshold depends on several parameters of the pump (wavelength, pulse length) and of the target (optical properties, melting or sublimation point) but is a crucial quantity for performing pump–probe experiments under controlled conditions. The damage threshold of silicon was studied under similar pump–probe conditions^13^ following previous works showing a clear dependence on the pump wavelength and pulse duration^13,29^ as well as a correlation between the dimensions of the damaged region and the deposited energy^30^. The damage threshold of GaAs irradiated by 70 fs pulses at 635 nm was determined previously^18^ but, to the best of our knowledge, no damage threshold studies are currently available for Ge and GaAs under pump–probe conditions similar to the present work.

## Methods and materials

The ultrafast reflectivity experiments were performed at the EuroFEL Support Laboratory (EFSL, ISM-CNR Rome) using a femtosecond laser system consisting of a chirped pulse amplifier (800 nm, 1 kHz, 4 mJ, 35 fs) seeded by a Ti:Sa oscillator. The pump pulse at 400 nm was produced by second harmonic generation (BBO crystal) and the white light supercontinuum used as a probe (340–780 nm) was generated by focusing 3 $\mu$J of 800 nm light into a rotating $\hbox {CaF}_2$ crystal. Those pulses are used within a commercial fast transient absorption spectrometer (FemtoFrame II, IB Photonics) in reflection mode. The optical layout consists in splitting (50/50) the probe beam into two with half reflected by the pumped sample and the remainder used as a reference. Both spectral intensities are measured for each pulse by separate detectors in order to minimize pulse to pulse fluctuations in the white light generation. The delay time between pump and probe was varied by changing the optical path of the probe pulses (high precision double pass linear stage). Hardware and software of the spectrometer are conceived to reduce the amount of data to be stored for such time-resolved experiments, and in our case data for the normalized reflectivity ${R(\lambda ,t)}/{R_0(\lambda )}$ were collected and arranged into an array for each time and wavelength in the selected time domain (typically from $-1$ to 300 picoseconds for present experiments). Further experimental details on the ultrafast spectroscopy setup can be found in previous works^8,11,31^.

The samples used in these pump–probe reflectivity experiments were $\sim$ 1 mm thick wafer single-crystals. Silicon was a (100) single-crystal lightly p-doped sample (dopant concentration $\sim 10^{16}$ $\hbox {cm}^{-3}$). Germanium was a (100) single-crystal undoped sample (intrinsic). Gallium arsenide was a (100) single-crystal sample (epiready n-doped). All these samples have been cleaned by acetone before measurements in order to remove possible impurities at surface. Reflectivity measurements were generally carried out at room temperatures and specific experiments at low temperature (80, 150 K) were also carried out for Ge using a liquid $N_2$ cooling device (Linkam THMS600).

## Results and discussion

### Pump–probe overlap and damage thresholds

**Figure 1:**
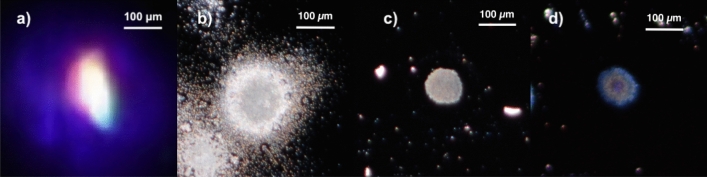
(**a**) Image of the pump and probe spots collected at Si(100) surface, correctly aligned at maximum overlap. (**b**, **c**, **d**) Nearly-circular damaged regions generated by pump pulses at 50 mJ/$\hbox {cm}^2$ ($\sim$ 1.7 $F_{th}^{Si}$), 26 mJ/$\hbox {cm}^2$ ($\sim$ 1.25 $F_{th}^{Ge}$) and 12.8 mJ/$\hbox {cm}^2$ ($\sim$ 1.33 $F_{th}^{GaAs}$) fluence for Si (**b**), Ge (**c**) and GaAs (**d**) respectively.

Present pump–probe measurements have been carried out for variable incoming pump fluences (single-shot energy for unit surface) measured by a calibrated power-meter. Real-time evaluations of the size of the focal spot and the overlap of pump–probe pulses were carried out using of a high-resolution CCD camera (Nikon D5200) equipped with a suitable long-distance macro optics (AF-S Micro Nikkor 105 mm f/2.8G IF-ED VR). The use of this device allowed us to collect images with a typical 10 $\mu$m resolution from a distance of about 30 cm. In Fig. 1a, we show a typical image for the size and overlap of the pump and probe (white) pulses. Image analysis of the profiles allowed us to evaluate the average pump spot size (diameter $\sim$ 150 $\mu$m FWHM). The typical size of the probe pulses at sample position resulted to be $\sim$ 100 $\mu$m wide (at maximum), enclosed within the pump spot as it is necessary for reliable transient reflectivity measurements.

The pump–probe overall overlap stability was verified at three different points of the linear stage (at the start, at the end and in the middle of the scan range). We remark that a stable pump–probe overlap is favored by the following features of the set-up: (1) limited excursion of the high-precision double pass linear stage of the delay line ($\sim 4.5$ cm for 300 ps delay) minimizing angular disalignments; (2) high reproducibility of the white light supercontinuum generation, positioned on the optical path of the 800 nm laser beam after the delay line so minimizing fluctuations in spectral probe profile in the entire travel range of the linear stage.

For all the experiments under consideration in this work, fluctuations of the pump–probe overlap were below our detection resolution (around 10 μm) so we can conclude that a stable and complete overlap between pulses was achieved.

As mentioned above, samples are permanently damaged for a fluence of the pump beam exceeding a well-defined threshold usually depending on the chosen photon wavelength, pulse time duration and on the sample material. Measuring the damage threshold $F_{th}$ for a given material and pump pulse is important for a smooth performance of multi-shot experiments measuring the transient reflectivity with high accuracy (typically over 10$^3$–10$^5$ shots). Moreover, it provides a natural scale for the deposited energy and the subsequent increase of electron and lattice temperatures in the pump-target interaction volume. Experiments to estimate the damage threshold were performed by increasing the incoming pump fluence (steps of $\sim$ 1 mJ/$\hbox {cm}^2$) for typical 1–10 s collection times (5$\times 10^2$–$10^3$ shots). Experimentally, the damage threshold corresponds to the maximum fluence for which experiments could be repeated at the same sample position without detectable changes of the signal. In this work, we have measured a fluence damage threshold for silicon of $F_{th}\sim$ 30 mJ/$\hbox {cm}^2$ with a typical 5% uncertainty, in nice agreement with previous results^13^. We have also measured for the first time the fluence damage thresholds at 400 nm for the Ge and GaAs single crystals under consideration. Those samples have been found to show much lower damage thresholds as compared to silicon, as expected in view of the larger optical absorption at 400 nm in these systems. In particular, we have estimated fluence damage thresholds of 20.8(8) mJ/$\hbox {cm}^2$ and 9.6(6) mJ/$\hbox {cm}^2$ for Ge and GaAs respectively. The present value for the damage threshold in GaAs results to be one order of magnitude lower than that measured for 635 nm pulses (100 mJ/$\hbox {cm}^2$) in a previous work^18^, confirming the important dependence of this quantity upon the excitation wavelength (observed in Si^13^ and mainly due to the increased absorption at lower wavelengths for a given pulse width).

We have verified by optical microscopy that the damaged regions of the sample increase their size increasing the incoming fluence beyond the threshold, in line with the results reported in Refs.^13,30^. In Fig. 1 we show images of the pump damage at the sample surface collected using the optical microscope in reflection mode for Si, Ge and GaAs. The large-sized damage observed in silicon (size of about $\sim$ 200 $\mu$m) was generated by a 50 mJ/$\hbox {cm}^2$ ($\sim$ 1.7 $F_{th}^{Si}$) pump fluence for 10 s. Smaller nearly-circular spots ($\sim$ 100 $\mu$m) were found for Ge and GaAs surfaces as shown in Fig. 1 at 26 mJ/$\hbox {cm}^2$ ($\sim$ 1.25 $F_{th}^{Ge}$) and 12.8 mJ/$\hbox {cm}^2$ ($\sim$ 1.33 $F_{th}^{GaAs}$) fluence respectively.

### Transient reflectivity in Si, Ge, GaAs: general features

**Figure 2:**
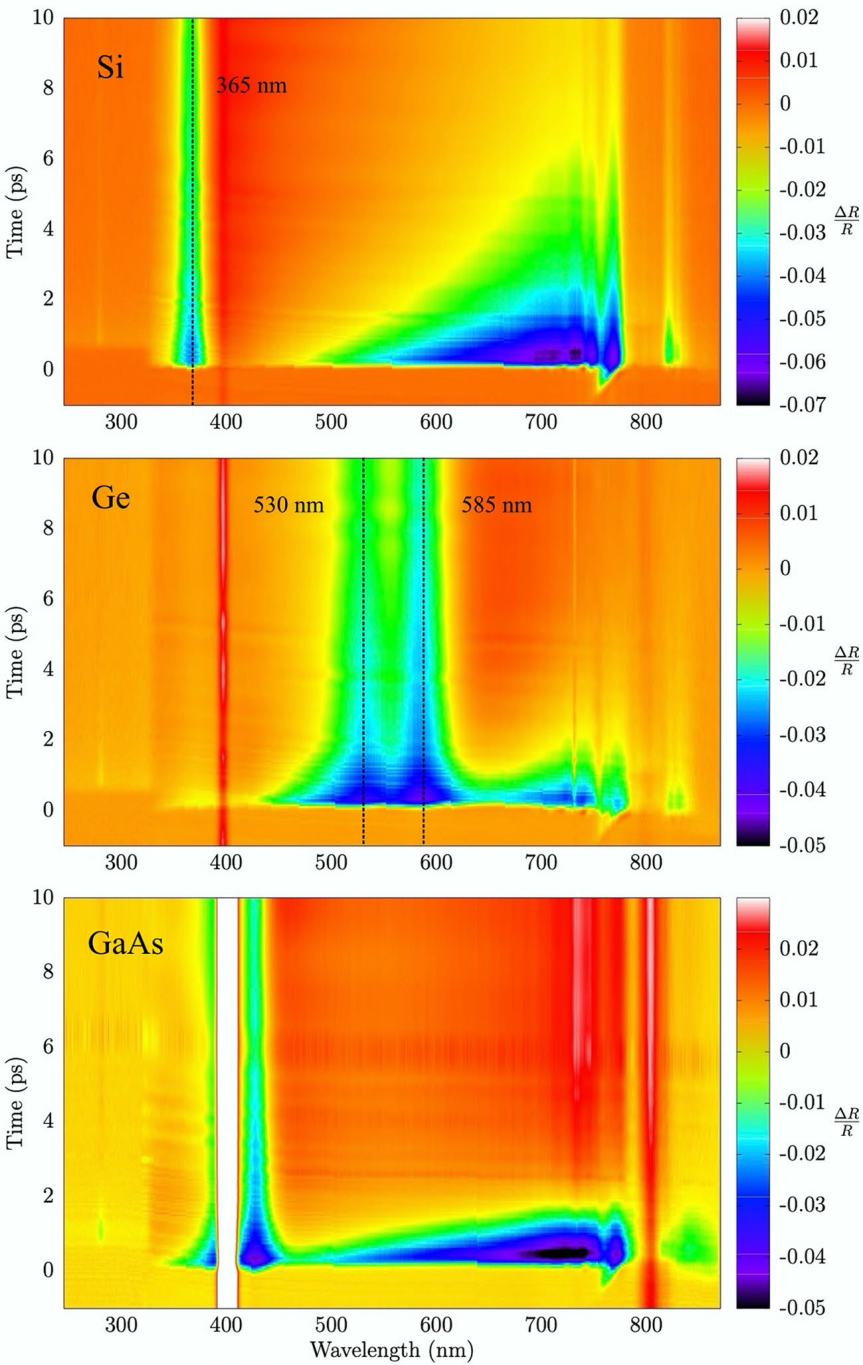
Transient reflectivity spectra of silicon, germanium and gallium arsenide for a pump fluence of 16 mJ/$\hbox {cm}^2$, 16 mJ/$\hbox {cm}^2$ and 6.4 mJ/$\hbox {cm}^2$ respectively.

Measurements of the transient reflectivity change $\Delta R /R = [R(\lambda ,t) -R_0(\lambda ))]/R_0(\lambda )$ (where $R_0$ is the unperturbed static reflectivity) have been then collected and pre-analyzed for typical fluences below the damage thresholds, removing chirp effects using standard procedures with a typical 100 fs accuracy. The measured transient reflectivity of our samples can be represented by a 2D map where the colors correspond to $\Delta R/R$ values for given probe wavelenghts (x axis) and pump–probe delay times (y axis), as shown in Fig. 2 for Si, Ge and GaAs (top to bottom) for comparable values of pump fluences and time delays up to 10 ps. However, present reflectivity data around 400 nm and 800 nm wavelengths are not fully reliable being near to the fundamental or doubled laser emission line so our detailed discussion in the following will be limited to the 450$\div$750 nm range.

**Figure 3:**
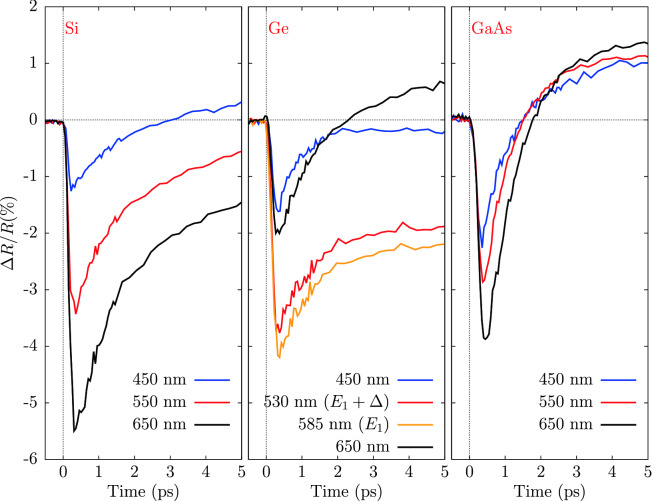
Time evolution of $\Delta R/R$ for Si (left panel), Ge (center panel) and GaAs (right-hand panel). Probe wavelength are 450 nm, 550 nm and 650 nm for Si and GaAs, while for Ge $E_1$ and $E_1+\Delta$ transitions wavelength instead of 550 nm. Pump fluences were 16 mJ/$\hbox {cm}^2$, 16 mJ/$\hbox {cm}^2$ and 6.4 mJ/$\hbox {cm}^2$ for Si, Ge and GaAs respectively.

Looking at Fig. 2, it is evident that silicon (upper panel, fluence of 16 mJ/$\hbox {cm}^2$, about 53% of the damage threshold) shows different dynamical regimes depending on the wavelengths of the probe. Firstly, we notice a long lifetime perturbation in reflectivity around 365 nm corresponding to the $E_1$ singularity in silicon at 3.40 eV and indicated by the vertical line in the figure. On the other hand, an increasing $|\Delta R/R|$ is obtained for Si (becoming more negative at short delay times as shown in Fig. 3) as the probe wavelength increases in the 450$\div$750 nm region. The recovering of the original reflectivity results to be quicker for shorter wavelengths. These results are in agreement with previous observations (see for example^4^ and refs. therein).

The $\Delta R/R$ spectrum of crystalline germanium (fluence of 16 mJ/$\hbox {cm}^2$, about 77 % of the damage threshold) is shown in Fig. 2 (center panel). The transient reflectivity change results to be dominated by two clear features: the first around 585 nm (2.12 eV) and a second one around 530 nm (2.34 eV) (see vertical lines in Fig. 2). These energies correspond to $E_1$ and $E_1+\Delta$ transitions of germanium at 300 K, as discussed in Refs.^21,25,26^. On the other hand, for probing wavelengths in the 620–760 nm range, germanium transient reflectivity is more similar to that of silicon (see also Fig. 3).

The trend of transient reflectivity of GaAs (fluence of 6.4 mJ/$\hbox {cm}^2$, about 66 % of the damage threshold, Fig. 2, lower panel) is qualitatively similar to the other two materials, but in this case the $E_1$ and $E_1+\Delta$ transitions^25,26^ are superimposed to the pump laser wavelength (400 nm). On the other hand, in the 450$\div$750 nm range, the transient reflectivity changes sign from negative to positive in less than $\sim$1–2 ps, more rapidly as compared for example with silicon (see also Fig. 3). Moreover, $\Delta R/R$ equilibrates (to zero) rapidly for shorter wavelengths (lower than $\sim$ 500 nm) while shows longer lifetimes increasing the probing wavelength.

The peculiar behaviour of the optical transient reflectivity near the $E_1$ singularities in Si, Ge, and GaAs is clearly observed in present experimental data of Fig. 2 and compare well with similar results obtained in transient absorbance measurements of ZnSe and Si nanowires^8,11^.

### Temporal evolution of transient reflectivity

It is interesting to look into the details of the temporal evolution of the transient reflectivity for selected probe wavelengths, as shown in Fig. 3 for the case of silicon (left panel), germanium (center panel) and gallium arsenide (right-hand panel). For better visibility, data of Fig. 3 are shown for a limited range of delay times 0–5 ps at selected wavelengths (for Si and GaAs 450 nm, 550 nm and 650 nm; for Ge we report the $E_1$ and $E_1+\Delta$ wavelengths instead of 550 nm) and for intermediate values of pump fluences below the damage thresholds. The transient reflectivity at very short delay times of Si, Ge and GaAs looks quite similar showing an abrupt decrease just after the pump pulse with a minimum value strongly dependent on the probe wavelength. However, while for Si and GaAs the negative variation is larger as the probe wavelength increases, for germanium the maximum of $|\Delta R/R|$ is reached for wavelengths in the 500$\div$550 nm range, corresponding to the $E_1$ and $E_1+\Delta$ energy interval^25,32^. Present silicon transient reflectivity spectra at these probe wavelengths are in nice agreement with previous results^4,7,13^ while no directly comparable data are available for Ge and GaAs. Present data for GaAs are globally in line and complete previous experiments^5,17,18^ obtained under different pump–probe conditions.

A detailed interpretation of the temporal evolution of the transient reflectivity is beyond the scope of the present work, however the trends can be interpreted in the framework of the model previously applied to silicon^4,6,7^, so considering the three contributions due to the injection of free carriers, the effect of state filling and of the lattice heating as a consequence of electron-phonon energy exchange.

**Figure 4:**
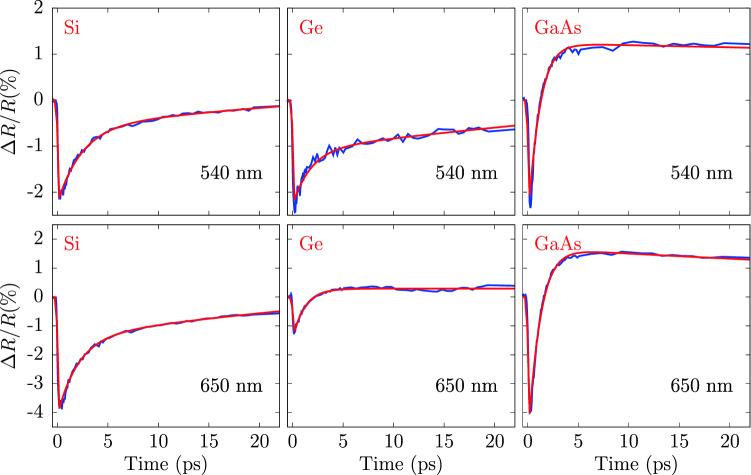
Time evolution of $\Delta R/R$ for Si (left panel), Ge (center panel) and GaAs (right-hand panel) at selected wavelenghts. Experimental data (blue curves) are compared with the results of a simple exponential model. Probe wavelength are 540 nm (upper panels) and 650 nm (lower panels). Pump fluences were 16 mJ/$\hbox {cm}^2$, 16 mJ/$\hbox {cm}^2$ and 6.4 mJ/$\hbox {cm}^2$ for Si, Ge and GaAs respectively.

Within this approach, the ultrafast transient reflectivity has been modeled by considering two main contributions, in which the first one ($\Delta R_N$) is intended to incorporate both free carrier and state-filling contributions, and it is assumed to be proportional to the (time) variation of the number of excited carriers ($\Delta N$) for a given fluence *F* and wavelength $\lambda$:

1 $$\begin{aligned} \Delta R_N=-C_N\cdot \Delta N(t)\cdot \lambda ^2. \end{aligned}$$

In this equation, the main wavelength dependence, $\lambda ^2$, is directly related to the free carrier contribution to the dielectric constant calculated in a Drude-like approximation.

The crucial quantity appearing in Eq. (1) is the decay of the number of free carriers $\Delta N(t)$ following the initial pump excitation. In analogy with the case of crystal silicon^4,7^, we can use a simple exponential model

2 $$\begin{aligned} \Delta N(t)=\frac{e^{-t/\tau _1}+C\;e^{-t/\tau _2}}{1+C} \end{aligned}$$

described by two time constants: $\tau _1$, related mainly to electron-phonon energy exchange and $\tau _2$, which can be associated with other relaxation processes. *C* defines the weight of the two processes in the decay.

The second contribution in the transient reflectivity is related to the time and fluence-dependent lattice temperature variation $\Delta T_L(t,F)$ and can be written as:

3 $$\begin{aligned} \Delta R_T=C_{term}\cdot \frac{dR}{dT}(\lambda )\cdot \Delta T_L(t,F), \end{aligned}$$

where *dR*/*dT* is the temperature derivative of the reflectivity and $C_{term}$ is a suitable constant that may depend on the incoming fluence and considered material. The time evolution of the lattice temperature variation $\Delta T_L(t,F)$ appearing in Eq. (3) is also naturally associated with the typical electron-phonon energy exchange time constants $\tau _1$ and $\tau _2$, increasing the lattice temperature, and with much slower equilibration times of the lattice temperature (thermalization to ambient temperature). Typical equilibration times are much larger ($\sim$ 200–1000 ps) than the ultrafast electron relaxation processes (0.2–10 ps) and are not considered here.

The simple phenomenological model for the carriers relaxation described above is able to reproduce the transient reflectivity change of the present experiments (full details and results for the different parameters and experimental conditions are given in Ref.^33^). An example of the application of the model to Si, Ge, GaAs transient reflectivity data is shown in Fig. 4 for selected wavelengths in the range 0–20 ps. In particular, the time constants are found to be smaller in GaAs ($\tau _1 \sim$ 0.5 ps, $\tau _2 \sim$ 1 ps) as compared to Si ($\tau _1\sim$ 1 ps, $\tau _2 \sim$ 50 ps) and Ge ($\tau _1\sim$ 1 ps, $\tau _1\sim$ 10 ps) in the wavelength range 450$\div$750 nm. However, peculiar phenomena take place near the $E_1$ singularity in Ge. As shown in Figs. 2 and 3 , the transient reflectivity change of Ge shows a long lifetime negative perturbation at wavelengths lower than $\sim$ 600 nm. The slow recovery is especially visible in the $E_1$, $E_1+\Delta$ range (530 nm and 585 nm) as shown in the center panel of Fig. 3. These finding are in line with recent femtosecond pump–probe ellipsometry measurements^21^ of the Ge dielectric constant. In present measurements, the negative reflectivity change of germanium in the vicinity of the $E_1$,$E_1+\Delta$ range is observed to survive up to the maximum delay times under investigation (up to $\sim -$0.5% at 300 ps delay times). The strong persistent negative transient reflectivity is associated with lattice heating, because the temperature derivative of the reflectivity at those wavelengths is negative^34^. On the other hand, the transient reflectivity $\Delta R/R$ of GaAs shows a very different behaviour, as compared to Si and Ge. In particular, $\Delta R/R$ changes sign becoming positive at very short delay times 1$\div$2 ps, while in silicon and germanium this bipolar behaviour is limited to certain wavelengths and for larger time delays, and is associated with lattice heating. However, the trend of present $\Delta R/R$ GaAs data is generally consistent both with previous observations^5,18^ who related the sign of the transient reflectivity to the temperature of the excited carriers, and with more recent studies discussing the ultrafast bipolar change in terms of band filling and band-gap renormalization effects^20^.

**Figure 5:**
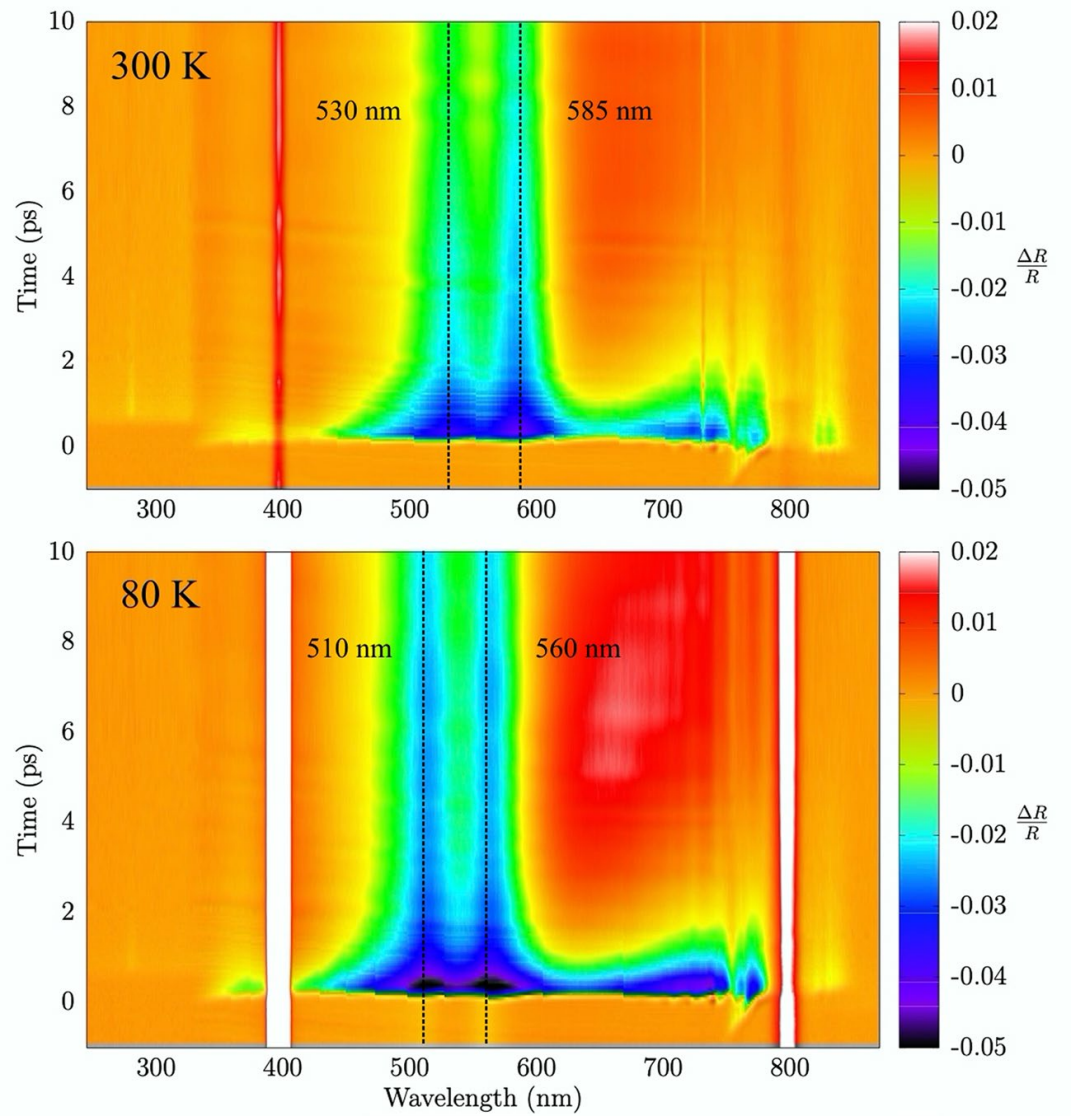
Transient reflectivity spectra of germanium measured at fluence of 16 mJ/$\hbox {cm}^2$ and at different initial temperatures of the lattice.

### Low-temperature Ge transient reflectivity

As mentioned above, for germanium we have also collected transient reflectivity measurements at low temperatures. The measured $\Delta R/R$ for Ge at 80 K and 300 K are compared in Fig. 5 at the same pump fluence (16 mJ/$\hbox {cm}^2$). The vertical lines depicted in that figure indicate the wavelengths of the $E_1$ and $E_1+\Delta$ transitions according to Ref.^25^.

**Figure 6:**
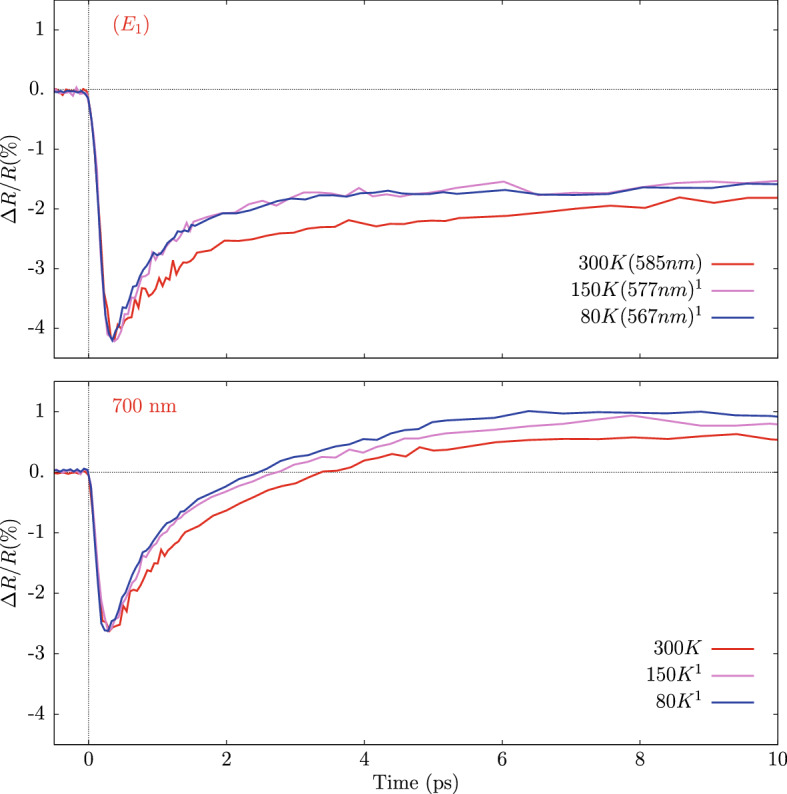
Transient reflectivity spectra of germanium (fluence 16 mJ/$\hbox {cm}^2$) collected at different sample temperatures and at selected wavelengths: those corresponding to the $E_1$ singularity (top) and at 700 nm (bottom). $^1$: normalized to the minimum $\Delta R/R$ value at 300 K.

The two maps of Fig. 5 show essentially the same features but with some notable differences associated with the temperature change. First, the main reflectivity features (vertical lines associated with the $E_1$ and $E_1+\Delta$ transitions) shift to lower wavelengths at lower temperatures ($\sim$ 20 nm at 80 K). The blue shift is confirmed also by another measurement at 150 K and we can estimate the average temperature rate for the $E_1$ doublet: $\Delta {E_{1 (doublet)}}/\Delta T=-(3.2\pm 0.6)\times 10^{-4}$ eV/K ($\Delta \lambda _{E_{1 (doublet)}}/\Delta T=(0.08\pm 0.01)$ nm/K), which is in substantial agreement with the rate reported in Ref.^25^ from static measurements: $-(4.2\pm 0.4)\times 10^{-4}$ eV/K.

In Fig. 6 we report the transient reflectivity of germanium at different temperatures for selected wavelengths up to 10 ps. Variations of the temporal dependence are observed at lower temperatures. In particular, the recovery times at 80 K and 150 K of the transient reflectivity shown in Fig. 6 are clearly shorter than the one at 300 K in the whole visible range, including the wavelength regions around $E_1$ (left panel) and those far from $E_1$ (right-hand panel, curve collected at 700 nm). We estimate a typical 30% reduction ot the time constants ($\tau _1\sim$ 0.7 ps at 80 K) resulting from the simple time evolution model discussed above. This reduction can be related to the lower electron density of the conduction band at low temperatures favoring relaxation and a more efficient equilibration of high-energy electrons/holes. Full explanation for the observed trend is however still under investigation.

## Conclusions

In conclusion, new ultrafast optical reflectivity experiments on silicon, germanium and gallium arsenide single-crystals have been presented in this work. Accurate measurements of the damage threshold in those systems excited by 400 nm and 35 fs HWHM pump pulses have been obtained and trends of transient reflectivity of Si, Ge, and GaAs have been studied for fluences below the corresponding threshold values. The large interval of probed wavelengths allowed us to comparatively study the ultrafast response of the three investigated materials in different photo n energy domains and clear relationships with the band structure affecting the transient reflectivity in selected wavelength regions. The temporal evolution of the transient reflectivity has been interpreted using a simple phenomenological model showing clear changes as a function of the probe wavelengths for the three semiconductors under consideration. Ultrafast optical measurements have been extended for Ge at low temperatures (down to 80 K) showing a clear shift of the relevant $E_{1_{(doublet)}}$ features and a modification of the time response. The present homogeneous set of experimental results on different simple materials may inspire theoretical works that could supply a unified picture of transient reflectivity in semiconductors.

## Data Availability

The data that support the findings of this study are available from the corresponding author upon reasonable request. http://gnxas.unicam.it/~dicicco.
